# Deeply Sequenced Infectious Clones of Key Cassava Begomovirus Isolates from Cameroon

**DOI:** 10.1128/MRA.00802-20

**Published:** 2020-11-12

**Authors:** J. Steen Hoyer, Vincent N. Fondong, Mary M. Dallas, Catherine Doyle Aimone, David O. Deppong, Siobain Duffy, Linda Hanley-Bowdoin

**Affiliations:** a Department of Ecology, Evolution, and Natural Resources, Rutgers University, New Brunswick, New Jersey, USA; b Department of Biological Sciences, Delaware State University, Dover, Delaware, USA; c Department of Plant and Microbial Biology, North Carolina State University, Raleigh, North Carolina, USA; Portland State University

## Abstract

We deeply sequenced two pairs of widely used infectious clones (4 plasmids) of the bipartite begomoviruses African cassava mosaic virus (ACMV) and East African cassava mosaic Cameroon virus (EACMCV). The ACMV clones were quite divergent from published sequences. Raw reads, consensus plasmid sequences, and the infectious clones themselves are all publicly available.

## ANNOUNCEMENT

Infectious clones are a central tool of molecular virology. Circular single-stranded DNA viruses such as begomoviruses are often cloned in a two-step process to create partial tandem dimers containing two copies of the virus origin of replication, a configuration that enhances infection (1). Cloned isolates of African cassava mosaic virus (ACMV) and East African cassava mosaic Cameroon virus (EACMCV) provided conclusive proof of synergy between two major clades of cassava begomoviruses (2), which is a defining feature of the epidemic of mosaic disease that has devastated cassava production in sub-Saharan Africa (3, 4). Here, we announce new sequence resources for these frequently used clones, which confirm the EACMCV clones and clarify the identity of the ACMV clones.

Complete and accurate plasmid sequences considerably simplify the molecular analysis of infectious clones and the design of new constructs. To confirm the sequences of the four plasmids listed in Table 1, we grew transformed Escherichia coli DH5α cultures overnight at 37°C with ampicillin selection and purified each plasmid with the Qiagen plasmid maxi kit. Libraries were prepared from Covaris-sheared plasmid DNA in triplicate with the NEBNext Ultra II kit and sequenced on the Illumina NextSeq 500 platform in the 150-bp paired-end read configuration.

**TABLE 1:** GenBank and Addgene identifiers for the four previously described infectious clones and corresponding virus (monomer) sequences^*a*^

Virus segment	Data for viruses			Data for plasmids		
	GenBank accession no.	Segment length (nt)^*b*^	G+C content (%)	GenBank accession no.	SRA accession no.	Addgene ID
ACMV DNA-A^*c*^	MT858793	2,781	44.7	MT856193	SRX8853831	159134
					SRX8853832	
					SRX8853835	
ACMV DNA-B^*d*^	MT858794	2,725	40.8	MT856194	SRX8853836	159135
					SRX8853837	
					SRX8853838	
EACMCV DNA-A^*e*^	AF112354	2,800	45.1	MT856195	SRX8853839	159136
					SRX8853840	
					SRX8853841	
EACMCV DNA-B^*f*^	FJ826890	2,732	44.1	MT856192	SRX8853833	159137
					SRX8853834	
					SRX8853842	

a Six complete sequences are described for the first time here, whereas AF112354 and FJ826890 were previously described (2, 5, 6).

b nt, nucleotides.

c pBluescript II KS(+) ACMV DNA-A 1.4mer.

d pUC19 ACMV DNA-B 1.5mer.

e pBluescript II KS(+) EACMCV DNA-A 1.6mer.

f pSL1180 EACMCV DNA-B 1.6mer.

These plasmids have been described (2, 5, 6) but not fully sequenced, so we deduced sequence maps (including partial tandem dimer virus segment inserts) based on the restriction sites used for cloning. Reads were trimmed with Cutadapt v1.16 (7) and aligned to these sequences, listed in Table 1, with the Burrows-Wheeler Aligner MEM algorithm (BWA-MEM) v0.7.13 (8). Variants relative to each reference sequence were identified with SAMtools v1.8 (9) and VarScan v2.4.4 (10). We corrected each plasmid sequence and aligned reads to it a second time.

The EACMCV DNA-A and DNA-B clones had four and two single-nucleotide differences, respectively, relative to their corresponding sequences in GenBank (accession numbers AF112354.1 and FJ826890.1, respectively). Relative to the new sequences (in the standard coordinate system starting from the virus replication origin nick site), these differences were T139A, G161R, T181TC, and A206AC for DNA-A and T1671G and A2724AT for DNA-B. The consensus sequences of the ACMV clones, however, were 3.1% and 5.8% divergent from the sequences (GenBank accession numbers AF112352.1 and AF112353.1) originally reported by Fondong et al. (2), as calculated with Sequence Demarcation Tool v1.2 (11). This difference was not entirely unexpected, because of the parallel history of two sets of ACMV clones; infectious partial tandem dimer clones were made via restriction digestion/ligation from sap-inoculated Nicotiana benthamiana plants, whereas the monomer segment unit clones were cloned with PCR from the same original cassava field sample (2). We expect that these complete infectious clone sequences will be of great utility to the community, given that many follow-up publications (12–23) specifically referenced the related but nonidentical monomer sequences (AF112352.1 and AF112353.1).

**FIG 1:**
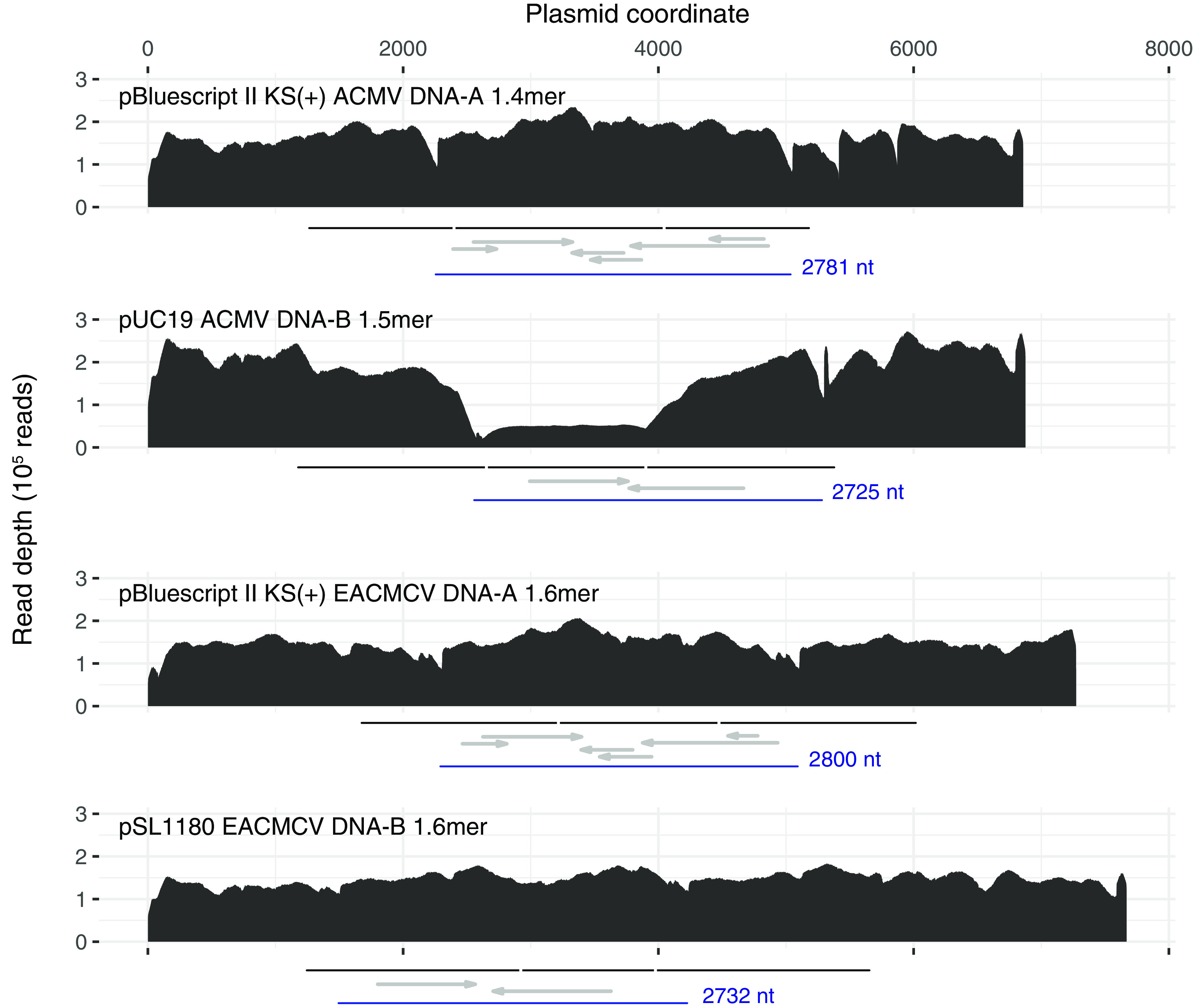
Plots of Illumina read depth across the length of the four infectious clone plasmids. One of three libraries is shown for each plasmid (Sequence Read Archive accession numbers SRR12354432, SRR12354427, SRR12354424, and SRR12354421). The region in each plasmid corresponding to each virus segment partial tandem dimer unit is indicated with a black line under each graph. Vertical white lines demarcate the boundaries of the unique and duplicated regions of each concatemer. Each virus segment monomer unit (between two replication origin nick sites) is shown in blue. Canonical virus genes are indicated with gray arrows, left to right for virus sense (AV1, AV2, and BV1) and right to left for complementary sense (AC1 to AC4 and BC1). The uneven read depth for the ACMV DNA-B plasmid is due to instability (truncation), which is evident in single-cut restriction digests (not shown). Such partial deletion of tandem duplicated regions in E. coli is not uncommon (24).

We obtained deep coverage, over 18,000-fold across all positions for all four plasmids, with an average of 157,000-fold coverage (Fig. 1). This read depth was consistent across three separate libraries for each plasmid and ensures the correctness of the partial tandem dimer sequences. Our results underscore the value of confirming the sequences of molecular clones.

### Data availability.

Plasmids are available from Addgene, and sequences for full plasmids and ACMV segments are available in GenBank (Table 1). The raw Illumina data are available from the NCBI Sequence Read Archive (PRJNA649777; Table 1). Data processing code has been archived as Zenodo record 4075362 (https://zenodo.org/record/4118067).
